# Recruitment Challenges and Strategies for Multimodal Intervention Trials

**DOI:** 10.1093/geroni/igab046.1144

**Published:** 2021-12-17

**Authors:** Jenna Bartley

**Affiliations:** Center on Aging, UConn Health, Farmington, Connecticut, United States

## Abstract

Determining ways to improve hip fracture recovery in older adults is important, however recruitment of this target population into clinical trials is challenging. Multimodal interventions that target multiple mechanisms of recovery may improve outcomes, but each component presents unique recruitment barriers. While exercise interventions have been shown to be beneficial for hip fracture recovery, offering exercise following completion of conventional physical therapy can be viewed as a burdensome time commitment. Hormone replacement therapy may hold promise for overcoming anabolic resistance, but concern about adverse side effects can also deter participation. STEP-HI is a multisite trial testing whether exercise and testosterone can improve hip fracture recovery in older women. In this talk, recruitment barriers experienced in STEP-HI and strategies employed to overcome these barriers will be discussed. Strategies include: partnering with hospitals, skilled nursing facilities and orthopedic surgeons. providing talks and education materials; and featuring past participant testimonials in recruitment materials.

